# Planning for the Strategic Recruitment of Barbershops for Blood Pressure Screening and Referral in the Mississippi Delta Region

**DOI:** 10.5888/pcd11.140179

**Published:** 2014-07-24

**Authors:** Vincent L. Mendy, Briana Perryman, Jackie Hawkins, Cassandra Dove

**Affiliations:** Author Affiliations: Briana Perryman, Jackie Hawkins, Cassandra Dove, Mississippi Delta Health Collaborative, Mississippi State Department of Health, Greenwood, Mississippi.

## Background

The 18-county Mississippi Delta region covers approximately 11,000 square miles of the northwest part of the state of Mississippi between the Mississippi and Yazoo rivers. In 2010, its population was 554,754; 49.7% of residents were black, and 46.9% were white (1). Heart disease and stroke are the second and fourth leading causes of death among black men in the region (2). In 2010, the heart disease death rate was 179.1 deaths per 100,000 in the United States (3), 251.1 deaths per 100,000 in Mississippi, and 259.4 deaths per 100,000 in the Mississippi Delta region (2). High blood pressure is a risk factor for heart disease and stroke and disproportionately affects black men (4); the prevalence of high blood pressure is higher among black men than among other racial/ethnic groups in the state (5). Efforts to reduce heart disease and stroke prevalence and disparities include prevention, early detection, and awareness of high blood pressure among high-risk and hard-to-reach populations. The Mississippi Delta Health Collaborative (MDHC) is a 5-year cooperative agreement between the Centers for Disease Control and Prevention (CDC) and the Mississippi State Department of Health designed to prevent heart disease, stroke, and related chronic diseases in the Mississippi Delta region. MDHC is implementing programs that target high blood pressure and hypertension prevention among at-risk populations in the Mississippi Delta region. One such program is the Barbers Reaching Out to Help Educate on Routine Screenings (B.R.O.T.H.E.R.S.) initiative. The goal of B.R.O.T.H.E.R.S. is to encourage barbers to routinely screen adult black men in the Mississippi Delta region, thereby increasing awareness of high blood pressure, and to refer clients with high blood pressure to a health care provider. Clients who are screened are provided resources on identifying local health care providers and education materials for promoting lifestyle changes and are encouraged to schedule routine checkups with a health care provider to monitor their overall health.

## Methods

From September 2012 through December 2013, in partnership with a local community-based organization (Mississippi Action for Community Education, Inc), barbers were recruited by MDHC through a request-for-proposal process and were trained to conduct blood pressure screenings and referrals at their barbershops. Fourteen barbershops were selected. Geographic information system techniques were used to map 1) the location of the barbershops, 2) the 2008–2012 age-adjusted death rates due to heart disease among black men by county, and 3) the 2010 population of black men by census block group level (6). Heart disease deaths were defined according to the following International Classification of Diseases, 10th edition, categories: I11, I13, and I20 through I51. Blood pressure readings were categorized as follows: normal blood pressure (systolic blood pressure <120 mm Hg and diastolic blood pressure <80 mm Hg), prehypertension (systolic blood pressure 120–139 mm Hg or diastolic blood pressure 80–89 mm Hg), and hypertension (systolic blood pressure ≥140 mm Hg or diastolic blood pressure ≥90 mm Hg) (7).

## Main Findings

The 14 participating barbershops are located in 10 cities. Among the 686 black men who received blood pressure screenings and referrals in the barbershops, 14.7% had normal blood pressure, 48.5% had prehypertension, and 36.4% had high blood pressure. Only 35% reported having a personal doctor, and only 43% reported having health insurance. Of the men screened, 34.3% were referred to a health care provider for follow up. The maps (Figure) indicate that many of the participating barbershops are located in counties with high rates of heart disease mortality and large populations of black men. However, the maps also show counties with high rates that have large populations of black men that lack barbershop participation (eg, Tunica, Quitman, Humphreys). The lowest county heart disease death rate in the Mississippi Delta region (236.4 per 100,000) is still substantially higher than the average for the United States (179.1 per 100,000) (3).

**Figure:**
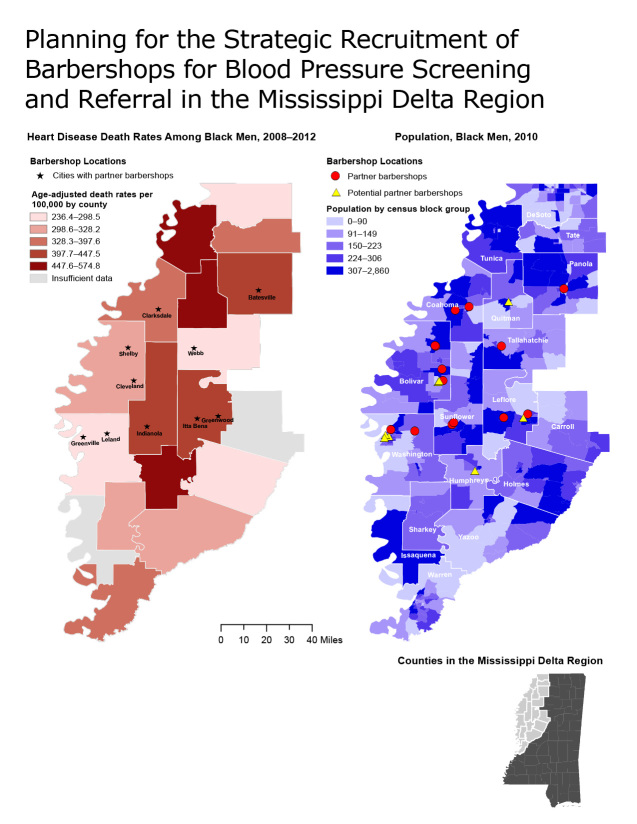
Planning for the strategic recruitment of barbershops for blood pressure screening and referral in the Mississippi Delta region. The maps show heart disease death rates by county and concentrations of adult black men at census block group level and locations of partner and potential partner barbershops. Together these maps help public health professionals identify communities that may be interested in improving hypertension awareness and treatment among black men by participating in the B.R.O.T.H.E.R.S. initiative to provide blood pressure screenings at barbershops. Heart disease is defined by the International Classification of Diseases, 10th edition, categories I11, I13, and I20 through I51 (2).

## Action

Our maps are designed to be used by faith-based communities, community leaders, mayors’ health councils, and community health workers in planning, building partnerships, recruiting, and encouraging barbers to conduct routine blood pressure screenings, particularly in counties with high heart disease death rates and population areas with a high concentration of black men. The map can also be used to evaluate placement of MDHC’s interventions and to plan for future recruitment of potential partner barbershops (barbershops that may become part of the initiative). MDHC aims to increase the number of barbers engaged in blood pressure screenings and the number of participants screened at each barbershop. In addition, MDHC plans to establish a provider network to better link participants with high blood pressure to local health care providers. Plans are under way to link participants with high blood pressure with community health workers to ensure that they visit their health care providers and navigate the health care system. Participants who do not have health care coverage will be encouraged to use the federal Health Insurance Marketplace to obtain coverage.
